# Microfabricated Polyacrylamide Devices for the Controlled Culture of Growing Cells and Developing Organisms

**DOI:** 10.1371/journal.pone.0075537

**Published:** 2013-09-24

**Authors:** Philippe Nghe, Sarah Boulineau, Sebastian Gude, Pierre Recouvreux, Jeroen S. van Zon, Sander J. Tans

**Affiliations:** FOM Institute AMOLF, Amsterdam, the Netherlands; University of California, San Diego, United States of America

## Abstract

The ability to spatially confine living cells or small organisms while dynamically controlling their aqueous environment is important for a host of microscopy applications. Here, we show how polyacrylamide layers can be patterned to construct simple microfluidic devices for this purpose. We find that polyacrylamide gels can be molded like PDMS into micron-scale structures that can enclose organisms, while being permeable to liquids, and transparent to allow for microscopic observation. We present a range of chemostat-like devices to observe bacterial and yeast growth, and *C. elegans* nematode development. The devices can integrate PDMS layers and allow for temporal control of nutrient conditions and the presence of drugs on a minute timescale. We show how spatial confinement of motile *C. elegans* enables for time-lapse microscopy in a parallel fashion.

## Introduction

The ability to create precisely controlled microenvironments has been pursued in microbiology [1], cell biology [2] and tissue engineering [3]. Microfluidic techniques have emerged as an important tool to impose spatial confinement, while allowing controlled delivery of nutrients and drugs, and drastically increase the level of parallel data acquisition [4,5]. Controlled delivery is important for observing cellular responses to external changes, but also to continuously replenish consumed compounds and depleting secreted waste that may become toxic. Realizing these features often requires complex device designs to separate the fluid flow from the observation chambers, involving multiple micro-structured layers, surface treatments and multiple modules [1-3,5], which can limit their applicability [6].

PDMS-based microfluidic devices are extremely versatile and have been applied to the culture of bacteria [7], yeast [8], mammalian cells [9], and even embryos [10] or nematode worms [11]. While allowing for exquisite control of flows, PDMS based devices often require sophisticated designs to achieve both confinement and controlled culture conditions and can ultimately be limited by the properties of PDMS as a material. PDMS devices can ensure localization of the object under study by confinement in micro-chambers and controlled medium exchange by laterally connecting channels that are narrow enough to prevent escape of the cells [12,13]. However, this typically requires multi-layered flow-cell designs and sometimes integration of *in situ* valves [14]. Watertight closure of the system is generally performed by plasma treatment of the PDMS [15], which can be incompatible with complementary treatments required to obtain the stable hydrophilic or hydrophobic properties ensuring appropriate adhesion of cells to the surfaces of the culture chambers [16]. PDMS is not permeable to aqueous solutions [15], which is desirable in some applications but a limitation in others, as it does not allow building osmosis or dialysis membranes, and can lead to local medium heterogeneities [17,18] and accumulation of toxic residues [19]. In addition, PDMS has poorly tunable mechanical properties, which are critical for the correct growth of many cell types [20,21].

Many of these issues could be addressed by the use of hydrogels. Hydrogels allow for free diffusion of the medium throughout the device, thereby ensuring uniformity of the cellular environment. In addition, hydrogels have highly tunable mechanical properties [22]. For these reasons, a variety of micro-environments based on hydrogels are being explored for tissue engineering [3,23]. So far, the most commonly used hydrogel for the study of micro-organisms as well as multicellular organisms is agarose, which is commonly used as an ‘agar pad’, a single monolayer of hydrogel, to support the growth of bacteria [24], yeast [25] or nematodes [26,27] in the imaging plane for live microscopy imaging. Simple layers of agarose have also recently been used as membranes to control bacterial medium in time [28-30]. In addition, agarose has been structured on the micrometer scale, e.g. to create grooves that guide the growth of bacteria [31,32] or to build microchambers to spatially confine live nematode larvae [33]. However, agarose is brittle and tears readily, making it difficult to manipulate, especially in the form of thin layers, which ultimately limits microfabrication possibilities ( [34], personal communication with the authors). In addition, agarose is composed of sugars and can be directly metabolized by some organisms [35] or may contain residual non-purified simple sugars, which could interfere with the study of growth under well-controlled conditions.

Here we propose polyacrylamide hydrogels as an alternative substrate for building controlled micro-environments. Polyacrylamide gels have several practical advantages that make them ideally suited for developing devices for live microscopy in biological studies. First, polyacrylamide is a commonly used material in biology laboratories for DNA and protein electrophoresis and its microfabrication requires minimal technological investments, as we will show below. Polyacrylamide gels are physico-chemically well-characterized and known to be biocompatible [36,37]. They are mechanically stronger (typical fracture energy *G* ~ 10-50 J.m^-2^ [38]) than agarose gels (*G* ~ 0.1-6 J.m^-2^ [39,40]) and hence allow for easier handling and are better suited for microfabrication. Their elastic properties are tunable over a wide range (from a fraction to several tens of kPa) by changing the total acrylamide content and the acrylamide to bis-acrylamide ratio [37], which allows for the construction of cell culture matrices with well-controlled mechanical properties [36,41,42]. They are permeable to aqueous solutions and composed of a synthetic polymer that cannot be metabolized as a carbon source, which allows for excellent control of the growth conditions. While polyacrylamide gels have been photopolymerized inside glass microchannels for *in situ* microfabrication [43], to build miniaturized electrophoresis devices [44,45] or inside PDMS channels to create flat cell culture matrices with elasticity gradients [46], their unique properties have not yet been exploited to construct micro-structured devices for biology studies using standard soft lithography techniques.

Here, we demonstrate two essential properties of polyacrylamide gels that enable the building of controlled environments and can be used simultaneously. First, we describe a soft lithography method to transfer micropatterns, such as confining culture chambers and microchannels, from a silicon wafer to a polyacrylamide gel. Second, we show how to use polyacrylamide gels as membranes to control the transfer of chemicals, building on previous designs using other materials that use dialysis membranes to change medium in time [47] or that use diffusion between lateral channels to generate gradients in space [34,48]. We describe several elementary designs, which consist of a polyacrylamide membrane constrained between glass or PDMS components, and demonstrate their use for the study of a range of organisms. We show that one can control the growth of *E. coli* bacterial colonies by controlling the carbon source as a function of time, and simultaneously track the response on the level of single-cell lineages. We also show that we can confine and grow *S. pombe* yeast cells, imposing a time-controlled reversible depolymerization of microtubules by a drug. Finally, we show that we can confine *C. elegans* nematode larvae in microchambers and follow the growth of multiple individual larvae in parallel by time-lapse microscopy.

## Results

### 1. Microfabrication of polyacrylamide gels

#### Master mold

Microfabricated polyacrylamide membranes can be indefinitely reproduced by soft-lithography from a single silicon wafer comprising the desired micropattern made of an epoxy resin, such as SU-8 (Methods). The initial master mold was made according to standard protocol by UV-lithography from a printed transparent mask, allowing the specification of an arbitrary two-dimensional pattern with a uniform height determined by the user, typically ranging from 1 to 100 microns.

#### Molding of the acrylamide

The aqueous solution of acrylamide monomers mixed with curing agents is poured on the master mold within a contour made of glass or metal of pre-determined height, bound to the wafer with silicon grease (Methods, Figure S1). The molding cavity is then closed with a silanized glass coverslip. A gel is generally obtained after 20 min at room temperature, but waiting 2 h ensures optimal polymerization. After polymerization, this results in a polyacrylamide gel with one face shaped as the negative of the micropattern of the master mold. We could easily obtain replica of molds going down to 10 micron features with an apparent fidelity at the micron scale (Figure S2). Note that we also use unstructured flat polyacrylamide membranes in the following. These are obtained according to the same protocol in which the wafer has been replaced by a silanized glass slide.

#### Preparation of the polyacrylamide membrane

After polymerization, the top silanized coverslip is removed, after which the gel is cut to the experimentally required dimensions and removed with tweezers. Importantly, the polyacrylamide membranes should be rinsed in water to remove unpolymerized acrylamide monomers, which are in particular known for their neurotoxicity. Proper rinsing has already been demonstrated to ensure biocompatibility of polyacrylamide matrices for neuron growth and development for weeks [49]. Transferring the polyacrylamide layer to fresh purified water at least two times for approximately 1 h proved sufficient to ensure the absence of observed growth defect in the organisms studied in this article. The membrane can be stored for several weeks in an aqueous solution without detectable degradation, consistent with quantitative studies of long term polyacrylamide degradation [50]. Before using the polyacrylamide membrane for a cell or organism culture experiment, we soaked it two times in the appropriate medium.

### 2. Experimental designs

**Figure 1:**
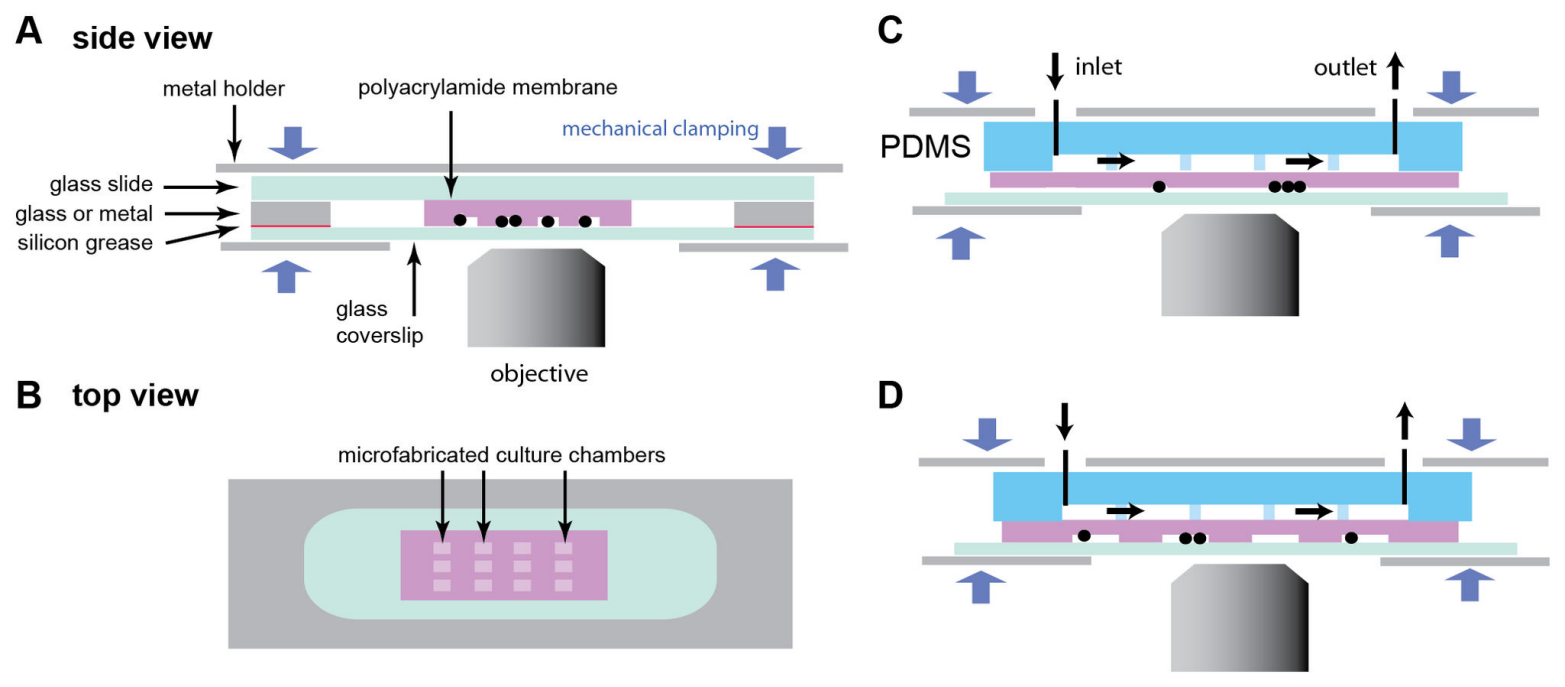
Schematics of devices for cells or organisms culture in polyacrylamide membranes. In all devices presented here, the different layers are held together by mechanical clamping, and the cells or organisms (represented as black circles) grow at the interface between the polyacrylamide gel and a glass coverslip through which microscopy is performed. (A) Side view of a microfabricated membrane comprising culture chambers, mechanically clamped between a glass slide and a glass coverslip. The device is sealed with a glass or metal contour. Sealing can be enhanced by adding vacuum silicon grease between surfaces. (B) Top view of panel A showing the array of microchambers surrounded by the glass or metal contour. (C) Flow cell using a PDMS control channel in contact with a polyacrylamide monolayer, which allows transfer of the flowing medium to the cells. In this design, cells are compressed underneath a flat polyacrylamide monolayer. (D) A more complex device combining the microchambers of the design in panel A and with the PDMS control channel in panel C.

In Figure 1, we first outline the different designs used in this research. In general, all devices consist of multiple layers stacked on top of each other, with the entire device being held together by mechanical clamping (Figure 1). In our case, this clamping was achieved by a metal holder with screws, with the appropriate openings for microscopy acquisition and microfluidic connectors.

In all different designs the objects under study are confined between the polyacrylamide membrane and the glass coverslip through which microscopy imaging is performed. Here, we use two different approaches to achieve this. In the first design, we enclose the membrane within a surrounding glass spacer sealed to a top glass slide with vacuum silicon grease (Figure 1A, B). This simple design ensures sufficient airtightness to limit evaporation and allows for observation under constant conditions for at least two days, provided that nutrients in the hydrogel membrane are present in excess. In the second design, a PDMS device containing a control channel is placed on top of and in direct contact with the polyacrylamide membrane (Figure 1C), thereby allowing continuous diffusion of the medium to cells or organisms growing below the hydrogel membrane. With this type of design, flow rates of a few tens of µL.min^-1^ allow one to switch the media within seconds, generating pressures below 100 Pa, allowing continuous use without leakage for at least a day. As the mechanical clamping used in all these designs avoids irreversible sealing or chemical bonding, the PDMS channel can be re-used many times.

Potentially, microfabricated polyacrylamide membranes and PDMS control channels can be combined depending on the experimental needs (Figure 1D). Possible designs are not limited to those in Figure 1: as an example we demonstrate below a device with channels embedded in the membrane.

### 3 Temporal and spatial control of the microenvironment

First, we used the advantageous transport properties of polyacrylamide gels to precisely control the medium composition in time, using an unstructured gel as a membrane, as well as in space, by setting up a concentration gradient within a membrane comprising molded microchannels.

**Figure 2:**
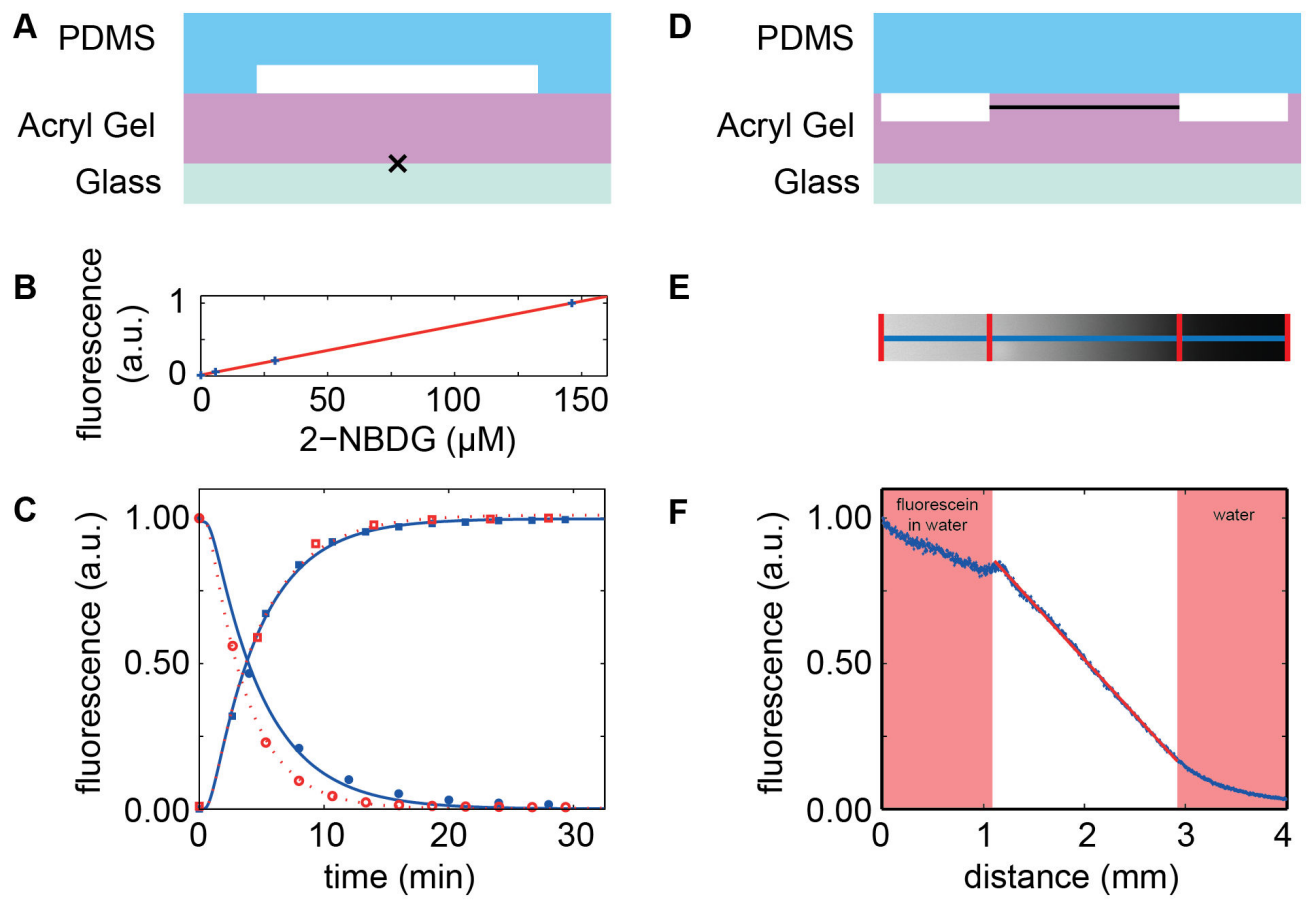
Diffusion in unstructured and structured polyacrylamide hydrogel membranes (A) Sketch of the flow cell device. An unstructured acryl gel (height 500 µm) is sandwiched between a PDMS layer comprising a channel (height 113 µm), and a glass coverslip, similar to the design in Figure 1A. (B) Fluorescence of the small dye 2-NBDG is proportional to its concentration in the flowing solution. (C) Fluorescence signal after infusion (squares) or depletion (circles) of the dye 2-NBDG was measured at the gel-glass interface (black cross in A). Lines show fits to the 1D diffusion equation. Open symbols and dashed lines correspond to flow rates of 50 µl.min^-1^, closed symbols and solid lines to flow rates of 20 µl.min^-1^. (D) Sketch of the linear gradient generator device. A structured polyacrylamide hydrogel (height 1 mm) is sandwiched between a PDMS layer and a glass slide. Water containing 3.5 µg.ml ^-1^ fluorescein is flown through the left channel, while pure water is flown through the right channel, thereby creating a linear concentration gradient within the gel. (E) Image of the fluorescence intensity profile at mid-channel height (black line in D) taken 85 min after the flows were established. Red lines indicate channel walls. (F) Fluorescence intensity profile (blue crosses) plotted versus distance (along the blue line in E). The fluorescence intensity in the acryl gel in between the channels is linear (red line).

In the first experiment, time control of the medium was obtained by placing a 500 µm thick gel membrane between a structured PDMS layer and a glass coverslip (Figure 2A). Flow was established in the PDMS channel with a syringe pump at flow rates ranging between 20 and 50 µl.min^-1^. A fluorescent glucose analog (2-NBDG) was added or removed from the flowing medium at a particular time point by switching a valve, thereby changing the composition of the flowing medium within seconds. The amount of 2-NBDG fluorescence was measured as a function of time by standard fluorescence microscopy using a 100X objective focused on the gel-glass interface. After the change of medium, the measured fluorescence signal rose in an approximately exponential fashion to the newly imposed steady-state value with a half-time of ~5 min (Figure 2B, C). Fits to the diffusion profile (Methods) yielded diffusion coefficients of 4.0x10^-10^ m^2^.sec^-1^ to 5.3x10^-10^ m^2^.sec^-1^, comparable to the typical diffusion coefficient of small molecules in water (~ 5.0x10^-10^ m^2^.sec^-1^).

In the second experiment, we aimed to set up a spatial concentration gradient by placing a structured gel membrane between a glass slide and a flat PDMS layer, the latter containing inlet and outlet connectors (Figure 2D). Liquid was pumped through the 100 µm high channels molded into the polyacrylamide hydrogel at 50 µl.min^-1^. One channel contained pure water, whereas the other contained an aqueous solution of fluorescein molecules. Diffusion of fluorescein into the polyacrylamide hydrogel, coupled with its removal at the adjacent channel, gave rise to a linear concentration gradient in the space between the two channels [34]. We found that the spatial gradient reached steady state after ~1 h. Subsequently, we imaged the concentration profile at mid-channel-depth. We observed a linear concentration gradient within the gel between the two channels as predicted by the theory (Figure 2E, F) [34].

### 4. Carbon controlled growth of bacteria

**Figure 3:**
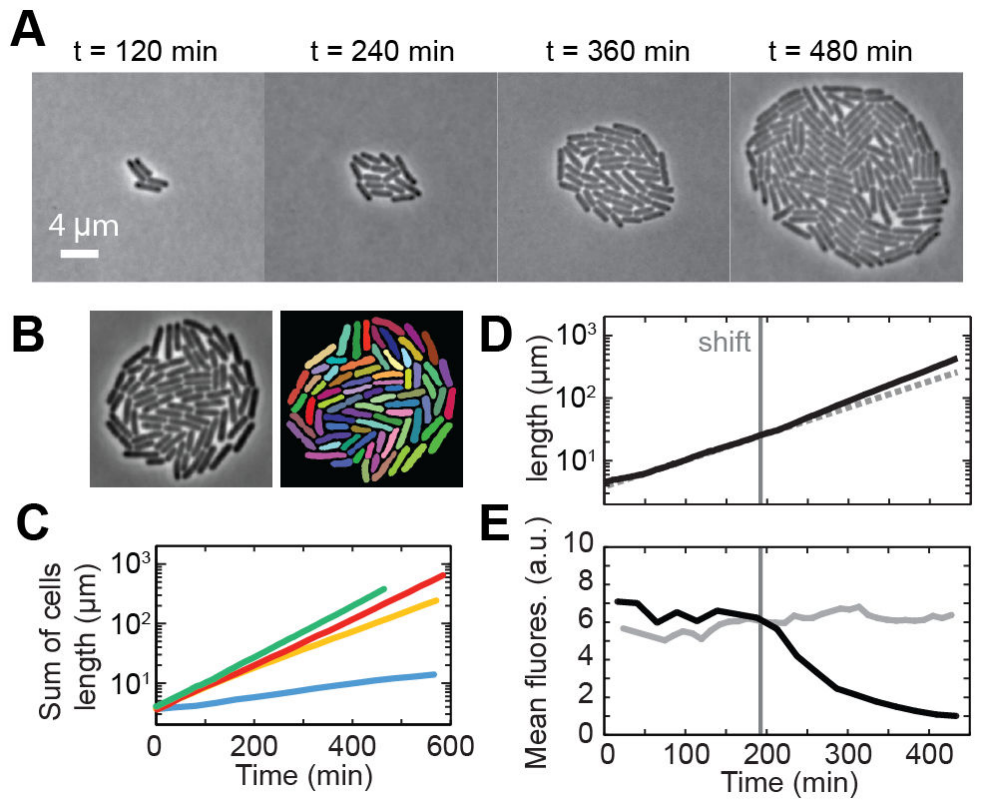
Monitoring bacterial growth by time-lapse microscopy. (A) Phase contrast images of *E*. *coli* cells growing in minimal medium supplemented with lactose. (B) Typical cell detection performed on a phase contrast image. (C) Sum of cells length for microcolonies growing on minimal medium with lactose (green), maltose (red), lactate (yellow) and limiting lactose (blue) as sole carbon source. (D) Sum of cells length during a shift from lactose to glucose and (E) corresponding mean fluorescence intensity of the colony. For comparison, fluorescence intensity for a colony growing only on lactose is shown in grey.

We tested the use of the polyacrylamide hydrogels to create a microfluidic chemostat for the growth of *E. coli* bacteria (Figure 1C). First, we used time-lapse phase contrast microscopy to visualize single *E. coli* cells growing on a simple flat polyacrylamide membrane while flowing minimal medium with abundant lactose. Cells divided for at least 8-9 generations into mono-layered colonies (Figure 3A, Movie S1). Given the absence of overlap between cells in the imaging plane, we could perform unambiguous determination of their outlines and determine the length of individual cells using custom image analysis software (Figure 3B, Methods). The population growth, quantified as the sum of the length of all cells in the microcolony, showed that cells were growing exponentially (Figure 3C, green trace) with a doubling rate of ~0.9 h^-1^.

Control of the growth rate of bacterial cells was demonstrated by exponential growth on minimal medium supplemented with various carbon sources (Figure 3C). The population growth showed that cells grew at a constant rate in each condition, yielding doubling rates of 0.8 h^-1^ for growth on maltose (Figure 3C, red trace), 0.6 h^-1^ on lactate (Figure 3C, yellow trace) and 0.23 h^-1^ on limiting lactose (Figure 3C, blue trace). These values are in good agreement with our growth rate measurements in bulk (Figure S3) and the relative quality of the different carbon sources ( [51] and Methods). Growth on limiting lactose confirmed in particular that the nutrient-free polyacrylamide matrix is suitable for attaining and studying low growth rates.

To show the ability of our designs to study cell dynamics in changing environments, we performed a carbon shift (with the device of Figure 1C as described in Section 3), and monitored both growth and gene expression over time. We started from a single cell on a minimal medium containing lactose, and switched to a minimal medium containing glucose after three generations. Expression of the *lac* genes was measured with a GFP reporter inserted in the lac operon (see Methods). The *lac* genes control the import and catabolism of lactose and are induced during growth on lactose, but repressed when glucose is present.

We show the population-level dynamics in Figure 3D-E. During growth on lactose, cells reached a steady state growth rate of ~0.8 h^-1^ and the mean fluorescence intensity of the microcolony per unit area over time was high, consistent with the full expression of the *lac* genes in all cells. Upon shifting to glucose, the growth rate was maintained at its pre-shift value during approximately 20 min, after which it increased abruptly to the higher glucose steady-state value of ~1 h^-1^. At the same time, the mean fluorescence started to decrease exponentially with a characteristic half time of 70 min, close to the doubling time. This indicated that upon the arrest of *lac* genes expression after the shift, the decrease in GFP intensity per unit area signal was dominated by dilution [52], until attaining cellular auto-fluorescence levels after four generations.

Overall, these experiments indicate that polyacrylamide gels allow for controlled and prolonged growth for constant nutrient conditions, and in response to a change in nutrient conditions.

### 5. Temporary depolymerization of microtubules in yeast by a drug

**Figure 4:**
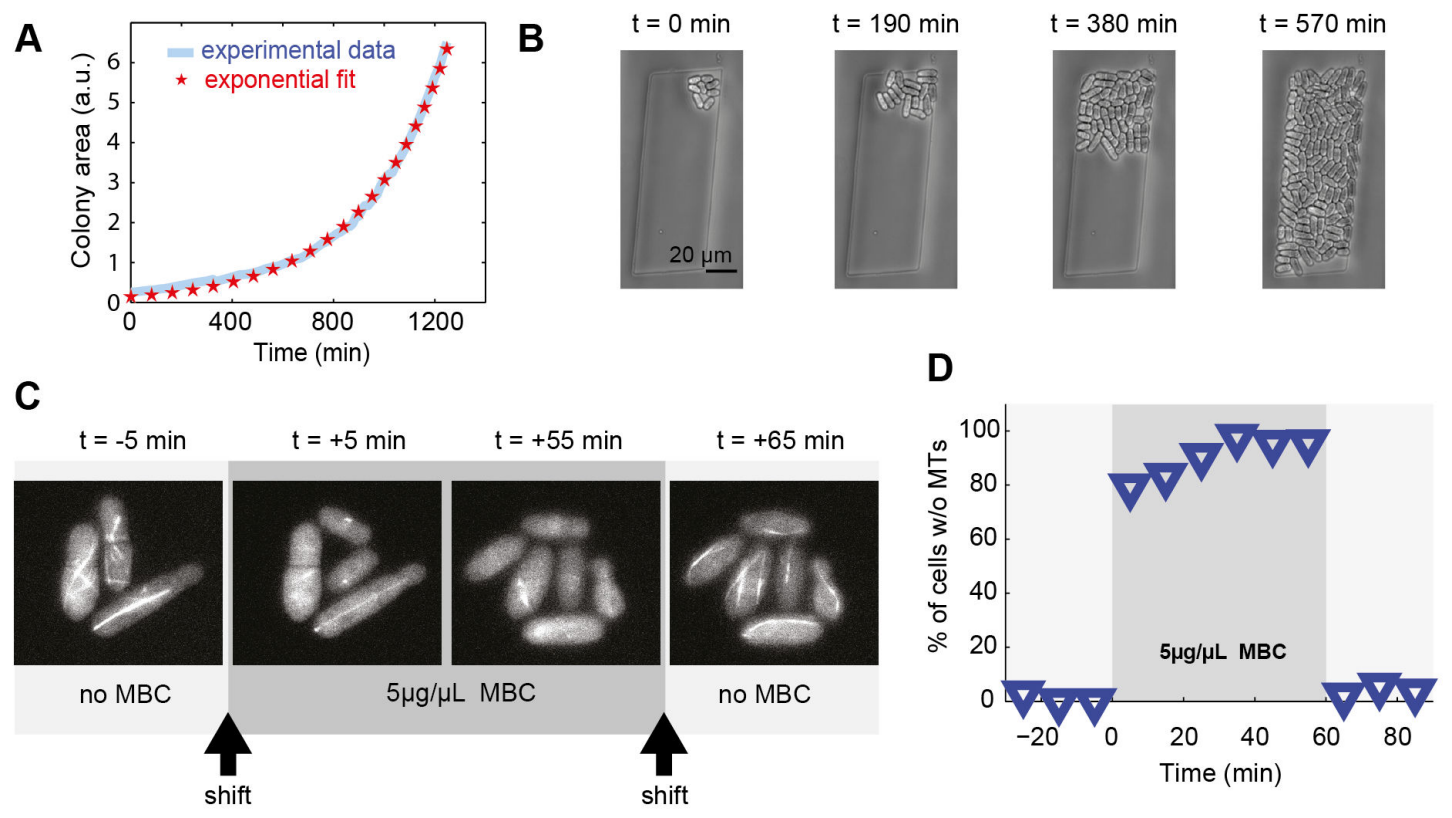
Control of the microenvironment of growing fission yeast colonies (A) Growth curve of the colony (area) obtained by a time-lapse experiment (blue) from 1 cell to 142 cells after 20 h (Movie S2). In red, single exponential fit of the growth rate with a doubling time of 240 min. (B) Phase contrast images of the fission yeast *S*. *pombe* growing in a microchamber (Movie S3) (C) Fluorescent microscopy images of fluorescently labeled microtubules during treatment with the microtubule-inhibiting drug methyl-2-benzimidazole-carbamate (MBC). Before the injection of 5 µM MBC, microtubules are observable in every cells (t = -5min). 5 min after the shift microtubules disappeared in more than 80% of the cells. MBC treatment lasts for 1 h, over which microtubule assembly is not observed. Rapidly after the wash out of the drug, microtubules reappeared in almost every fission yeast cell. (D) Percentage of cells without observable microtubules in a time-lapse experiment, before, during and after microtubule depolymerization with 5 µM of MBC.

To explore the application of polyacrylamide devices to observe fission yeast, we first investigated whether they grew normally, both between an unstructured hydrogel monolayer and a glass cover slip and confined within microstructures such as channels or chambers in a device similar to Figure 1A-B. When cells where positioned between an unstructured flat hydrogel and a glass coverslip, we could observe constant exponential growth of the fission yeast cells for more than 7 generations over 20 h (Figure 4A). This corresponded to an average doubling time of 170 min at 32°C, in agreement with liquid culture growth rate in the same minimal medium. This indicated that the mechanical pressure imposed by the hydrogel layer was soft enough to not perturb growth.

We then grew yeast cells, which have a 3-4 µm diameter, confined in 3 µm deep microstructures molded in the hydrogel. Time-lapse imaging showed that colony expansion was constrained by the walls (Figure 4B, movie S2).

We tested the ability of the microstructured membrane to control the chemical composition of the microenvironment, using a PDMS control channel on top (Figure 1D). Here we aimed to induce microtubule depolymerization with the drug methyl-2-benzimidazole-carbamate (MBC) during a certain time window. In fission yeast cells, microtubules form 3-5 bundles composed of groups of 2-4 microtubules. After growing yeast cells for 2 generations, we supplemented the flowing medium with 50 µM of the microtubule-destabilizing drug. Within 5 min, the fluorescently (GFP) labelled microtubules had disappeared in more than 80% of the cells (Figure 4C). In the remaining fraction of cells, abnormally short microtubules were present, as already reported for MBC-treated fission yeast cells [53]. After 60 min, the drug was removed from the flowing medium, leading to the reappearance of microtubules in more than 90% of cells within 5 min.

In conclusion, we have shown that yeast cells grow normally on polyacrylamide hydrogel membranes, and that colony expansion can be confined between a flat hydrogel membrane and a glass coverslip, or within a structured membrane. The membrane allowed for adding or removing a drug on a timescale of minutes.

### 6. Growth and development of spatially confined *C. elegans* larvae

**Figure 5:**
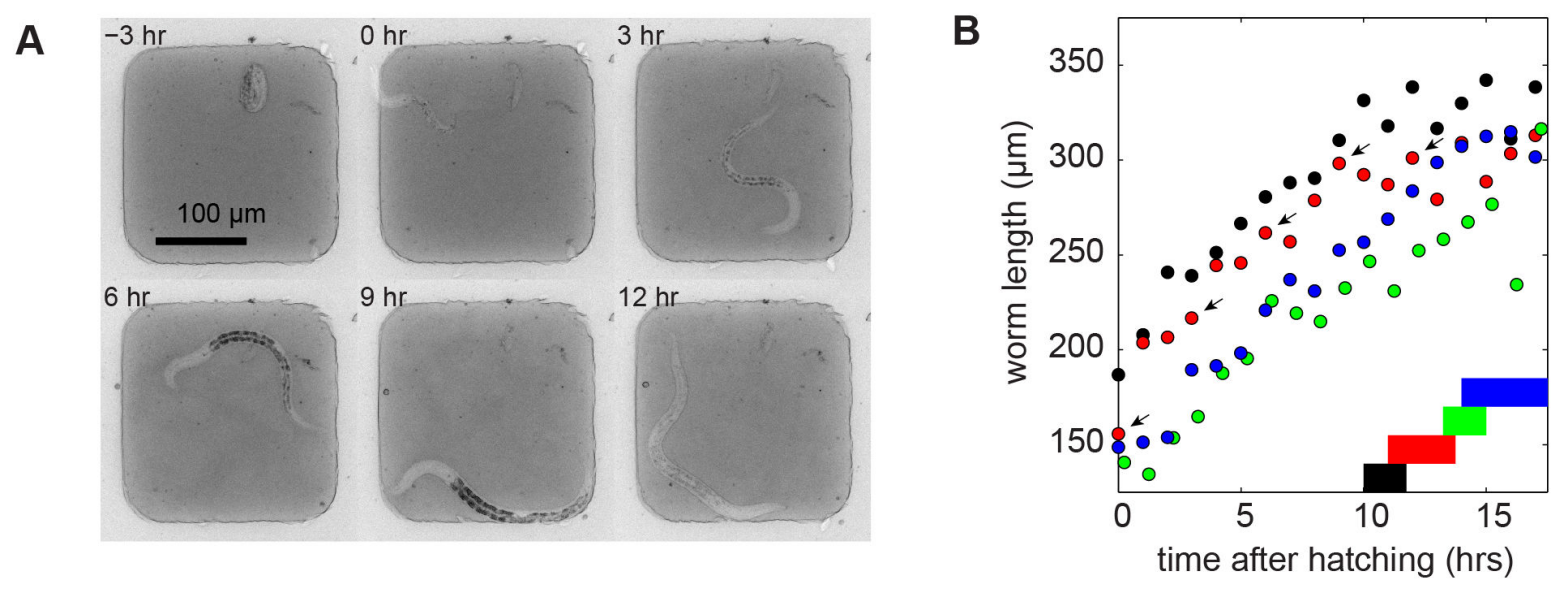
*C. elegans* growth in microchambers. (A) Growth of a single *C*. *elegans* animal through the L1 larval stage constrained in a 200 x 200 x 18 µm polyacrylamide microchamber filled with OP50 as source of food. Time is shown in hours after hatching. At 12 h after hatching the animal has entered the lethargus at the end of the L1 larval stage. (B) Worm length as a function of time after hatching. Different colors indicate animals grown in parallel on the same device. Horizontal bars show the duration of lethargus, ending with the molt at the start of the L2 larval stage. The markers indicated by the arrow correspond to the time points shown in (A).

Next, we aimed to investigate whether our devices can be used to monitor the growth and development of single larvae of the nematode worm *Caenorhabditis elegans*. Hence we enclosed *C. elegans* worms in an array of microchambers molded into polyacrylamide hydrogel (Figure 5A).

We positioned one *C. elegans* egg per microchamber together with *E. coli* bacteria as food source, and followed multiple chambers by time-lapse microscopy. The eggs were found to develop inside the polyacrylamide microchambers and newly hatched larvae increased in length from about 150 µm directly after hatching to about 350 µm over the course of 10-15 h (Figure 5A, 5B and Movie S4). Despite their active motility, all larvae stayed confined to the microchambers during the entire period of observation.

Development of *C. elegans* is divided in four larval stages, labeled L1 to L4, that are separated by molts during which a new cuticle is synthesized and old cuticle is shed. The molts are accompanied by a behaviorally quiescent state called lethargus. After 10-15 h, animals entered the lethargus accompanying the L1 molt (Figure 5B), agreeing well with the observed duration of the L1 larval stage of ~15 h [26]. We observed that the start of the L1 molt correlated well with animal length: the observed variability in the time of entry into the L2 lethargus was mostly due to variation in animal length at the time of hatching. These results indicate that the nematodes were able to develop to the L2 larval stage inside the microchambers.

## Discussion

We have demonstrated the microfabrication of structured polyacrylamide membranes by soft-lithography and used these to build controlled environments for the study of growing cells and organisms. While similar capabilities can be achieved with other techniques based on agarose gels or PDMS, our method has several practical advantages. Agarose layers can easily tear, while the mechanical strength of polyacrylamide allows easy handling of the gel during microfabrication steps and assembling the flow cell, and provides high success rates, reproducibility, and well-defined structural features. We found that polyacrylamide membranes down to 140 µm thickness could be used routinely. In contrast, we found that agarose layers of the same thickness and elastic modulus (~50 kPa for 1% agarose [39]) systematically broke down during the fabrication process. In comparison to PDMS-based devices, which often involve more complex multi-layer lithography to define feeder channels, our method is simple and requires minimal investment in materials and technological infrastructure. Our protocol for fabrication of polyacrylamide hydrogels is commonly used in biology laboratories for protein electrophoresis and involves preparation of a solution that polymerizes at room temperature. Once polymerized, these membranes remain functional for months when stored in solution. The microfabrication step relies on a silicon mold that can be re-used multiple times.

We have tested various device-designs which allow accurate spatio-temporal control of the environment. Control in time was achieved by diffusion of the medium through the permeable gel into culture chambers that are hence uncoupled from the medium flow. Diffusion coefficients of small molecules in the gel are close to their value in water, which combined with the possibility to build thin membranes resulted in a medium exchange with a characteristic time of 5 min. This response time is well-suited for many applications that require the control of the growth and gene expression dynamics in micro-organisms such as bacteria or yeast. We demonstrated the creation of linear concentration gradients between two continuously flowing solutions in channels embedded in a single gel layer. This method may be used to set up gradients that are steeper or have more complex two-dimensional patterns than flow-cells used so far. In these existing designs, diffusion between PMDS channels is indeed constrained to occur through an unstructured agarose layer in a third dimension [34]. We have demonstrated the potential of these devices to study dynamically the genetic or the morphologic responses to changes in growth medium or the addition of chemical inhibitors of cellular processes. In conclusion, polyacrylamide-based devices are well suited to study the response of diverse biochemical pathways to chemical perturbations. They are well suited for metabolism and growth studies as the polyacrylamide matrix is free of nutrients.

We found that bacteria and yeast grew in well-defined monolayers when covered with polyacrylamide, as is also observed for agar pads, thus allowing convenient microscopy and analysis at the single cell level. The soft confinement provided by the polyacrylamide membranes ensured localization of the colonies, while maintaining normal growth and morphological phenotypes without requiring the deposition of an additional soft layer on the glass [47]. In our case, experiments were stopped when the colonies developed a second layer. For slow-growing cells, the devices allow observation of exponential growth for over 2 days. Longer term experiments, such as used to study aging, would require one to adapt designs including washing channels (see 54,55 for PDMS and [32] for agarose realizations). In addition, polyacrylamide allows chemical modifications for the control of the micro-environment, enabling advanced capabilities such as the controlled release of bacteria [56] or patterned bio-functionalization [57].

The technology is also promising for the study of larger, multicellular organisms, such as *C. elegans*. Polyacrylamide gels with microchambers provide important advantages. First, they allow spatial confinement of these otherwise highly motile organisms, enabling time-lapse microscopy and parallel image acquisition without the use of anesthetic drugs [27] or automated tracking of individual animals [58]. Second, polyacrylamide hydrogels enable exchange of medium and waste products with the microenvironment of the animal.

Finally, the tunable mechanical properties of polyacrylamide hydrogels make them potentially useful for the culture of other cell types, given for example the exquisite sensitivity of mammalian cells to the mechanical properties of their support [20]. The potential to embed microfabricated polyacrylamide membrane in complex modular designs offers exciting opportunities to develop well-controlled environments for cell biology studies and tissue engineering.

## Methods

### Fabrication

Master molds have been realized on silicon wafers with spin-coated SU-8 epoxy resins (MicroChem) of different viscosities (models 2005, 2025 and 2100) resulting in heights of 3 µm for the yeast chambers, 18 µm for the worm chambers and 100 µm for the gradient assay. No specific wetting treatment was done to the surface of the wafer. For the polyacrylamide gel, we used a 29:1 ratio of acrylamide / bis-acrylamide (Bio-Rad) with a final concentration of 10%. Polymerization was initiated by the addition of 0.1% of ammonium persulfate (Sigma) and 0.1% of TEMED (Sigma). The mixture was poured in a mold consisting of a cavity made of a machined glass or aluminum slide of thickness varying between 150 µm and 1 mm, glued to the wafer or to a simple silanized glass slide with vacuum silicon grease. A silanized glass coverslip was deposited on top and the solution was left to polymerize for about 2 h. The polyacrylamide membrane was then cut and transferred in DI water for conservation. The PDMS (Dow Corning) channel was molded on a silicon wafer with SU-8 according to the protocol provided by the resin manufacturer (MicroChem) and consisted of a 113 µm high and 5 mm wide channel comprising pillars to ensure uniformity of the pressure applied on the polyacrylamide membrane. Mechanical clamping of the whole device was ensured by a home-made metal holder with 4 screws, comprising openings on the bottom for microscopy acquisition and on the top for illumination and the tubing. To ensure a good seal between the polyacrylamide and the PDMS layer, we made sure that the hydrogel and the PDMS had a contact area that extended at least 3 mm away from the edges of the control channel.

### Experimental devices

The flow was externally driven with syringe pumps (ProSense, NE-1000 and NE-300) connected to the microfluidics device by polyethylene tubing of 0.58 mm internal diameter (Smiths medical International Ltd.). When using a PDMS channel, the device was degased 1 h in low vacuum prior to flow to allow removal of trapped air bubbles by suction from the PDMS matrix. Switches were performed by a manual valve (Hamilton, HV 4-4). All experiments have been performed with an inverted epifluorescence Nikon microscope TE-2000 U embedded in a temperature controlled box.

### Bacteria

Growth experiments were performed using derivatives of *E. coli* MG1655 (*rph-1 ilvG- rfb-50*). To measure the expression of the *lac* operon, *lacA* was replaced with *GFPmut2* (kindly provided by M. Ackermann, ETH Zürich). Cells were grown in M9 minimal medium (47.7 mM Na_2_HPO_4_, 25 mM KH_2_PO_4_, 9.3 mM NaCl, 17.1 mM NH_4_, 2.0 mM MgSO_4_, 0.1 mM CaCl_2_) with 0.2 mM uracil, supplemented with 0.1% (w/v) lactose, maltose or lactate and 0.001% (w/v) lactose in the limiting case. Note that adding uracil compensates for intrinsic pyrimidine starvation of the MG1655 strain [59] and accounts for the typically 15% higher growth rates measured in our study compared to Beg *& al*[51]. . Cells were initially inoculated from glycerol stock in TY medium and grown until the OD > 0.02 and next diluted in the appropriate medium overnight. The following day, the overnight culture was diluted in the same medium (OD ~0.005) and transferred to the microfluidic chamber. 10 µL of culture were deposited on the polyacrylamide gel membrane and left to dry for about 2 min before the setup was assembled. Optionally, addition of a surfactant in the medium (Tween 20 at 10^-5^ volume fraction) allowed further enhancement of colony growth into a monolayer. All these steps and the experiment were performed at 37°C. Images were acquired with a 100X oil immersion objective (Nikon, Plan Fluor NA 1.3). Phase contrast and fluorescence images were analyzed with a custom Matlab algorithm derived from an algorithm of the Elowitz lab (Caltech) [60]. The instantaneous growth rate was determined by fitting the cell length over time to an exponential function.

### Yeast

For growth experiments, we used the *S. pombe* wild type fission yeast PT286 h- ade6-M216 leu1-32 ura4-D18. For the pad growth experiment, fission yeast cells were pre-grown overnight in Edinburgh Minimal Medium (EMM) liquid culture. 2 µL of 10x-concentrated culture were deposited on a polyacrylamide gel membrane that had been incubated in EMM medium. All these steps and the experiment were performed at 32°C. Cells were imaged through a 40X objective oil immersion lens (Nikon, NA=1). Colony area was measured with ImageJ (http://rsbweb.nih.gov/ij) software. For the drug shift experiment, the same culture conditions were used for a strain expressing GFP tubulin (DB 871 h90 nmt1-GFP-tub:lys1+ leu- ura-) and microtubule detection was performed visually. For the confined growth experiment, we used a YE5S medium and performed imaging with a 20X objective (Nikon, NA=0.5) at 37°C.

### Nematodes

The wild-type (N2) *C. elegans* strain was grown on NGM agar plates covered with *E. coli* OP 50 as food source, following standard protocols [61]. Before sample preparation, the polyacrylamide microchamber array was soaked overnight in M9. Under a stereomicroscope, OP 50 bacteria and a single embryo at the three-fold stage, between 550 and 840 min after fertilization [26], were transferred to each individual microchamber, using a worm pick to transfer bacteria and an eyelash attached to a Pasteur pipet to transfer eggs. Images of individual microchambers were captured every 15 min using a 10X Nikon objective (NA=0.30). L1 larvae hatched and developed at room temperature (22°C). Worm length was quantified as a function of time with a 1 h interval. Entry into and exit from the L1 molt was monitored by eye, based on the reduction in movement during lethargus, the decrease in contrast in the transparency of the animal’s body due to synthesis of the new cuticle and finally the shedding of the old cuticle.

### Fitting of diffusion coefficients in Figure 2C

Formula was taken from Crank [62]:

$$C=C_{0}(\sum_{n=0}^{N}{\left(-1\right)}^{n}erfc\;\;\left(\frac{\left(2n+1\right)\;l-x}{2\sqrt{Dt}}\right)+\sum_{n=0}^{N}{\left(-1\right)}^{n}erfc\;\;\left(\frac{\left(2n+1\right)\;\;l+x}{2\sqrt{Dt}}\right))$$

where *l* = 500 µm (width of system), *x* = 0 µm (position of measurement), *C* is concentration and *t* is time, values taken from Figure 2C. Fitting was performed with Matlab with *N* = 3. The maximum concentration *C*_*0*_ and the diffusion coefficient *D* were used as fitting parameters.

## Supporting Information

Figure S1 **Molding of the polyacrylamide gel and assembly of the device.** A) Step 1: a cavity is prepared, consisting in the silicon wafer with the photoresist pattern, reversibly assembled with vacuum grease to a glass or metal contour of desired height. Step 2: the acrylamide solution is injected with a pipette within the cavity. Step 3: A silanized coverslip is then added on top of the cavity and polymerization occurs at room temperature for 2 h. B) Photographs of a multi-layered device: separate parts (top) and assembled device (bottom).
(TIF)

Figure S2 **SU-8 pattern on a wafer and molded acrylamide gel.** A) Image of a silicon wafer with 3 µm high patterns in SU-8 photoresist. B) Structures shown in A have been molded in a polyacrylamide gel. Scale bars 100 µm. The smallest features are 10 µm wide.
(TIF)

Figure S3 **Growth rates in batch cultures.** Optical density at 550 nm normalized by the OD at t=0 (and shifted for clarity) versus time for batch cultures of MG1655 cells growing in minimal medium with abundant (0.1%) glucose (dotted line), lactose (green), maltose (red) and lactate (yellow) as sole carbon source. Exponential fits to the experimental data points (lines) yielded growth rates of 1.12 h^-1^ on glucose, 1.01 h^-1^ on lactose, 0.88 h^-1^ on maltose and 0.54 h^-1^ on lactate, comparable to those obtained for cells growing in the microfluidic device (see main text and Figure 3B and 3D).
(TIF)

Movie S1 (AVI)

Movie S2 (AVI)

Movie S3 (AVI)

Movie S4 (AVI)
