# Supplementary figures and images for: Microfabricated Polyacrylamide Devices for the Controlled Culture of Growing Cells and Developing Organisms
Source: PLoS One. 2013 Sep 24;8(9):e75537. doi: 10.1371/journal.pone.0075537 (PMC3782435; doi:10.1371/journal.pone.0075537)

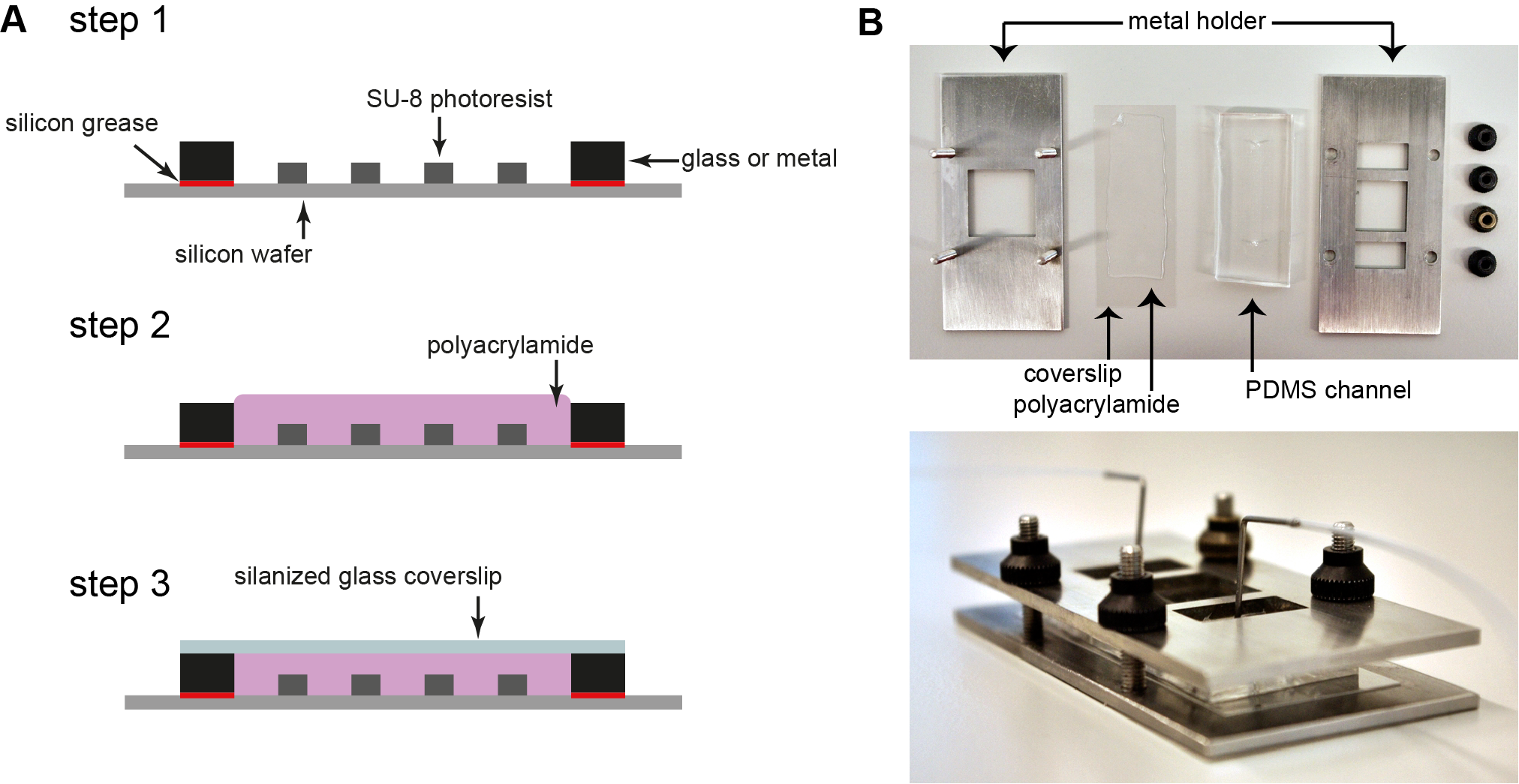

Supplement: Figure S1 — Molding of the polyacrylamide gel and assembly of the device. A) Step 1: a cavity is prepared, consisting in the silicon wafer with the photoresist pattern, reversibly assembled with vacuum grease to a glass or metal contour of desired height. Step 2: the acrylamide solution is injected with a pipette within the cavity. Step 3: A silanized coverslip is then added on top of the cavity and polymerization occurs at room temperature for 2 h. B) Photographs of a multi-layered device: separate parts (top) and assembled device (bottom). (TIF) [file pone.0075537.s001.tif]

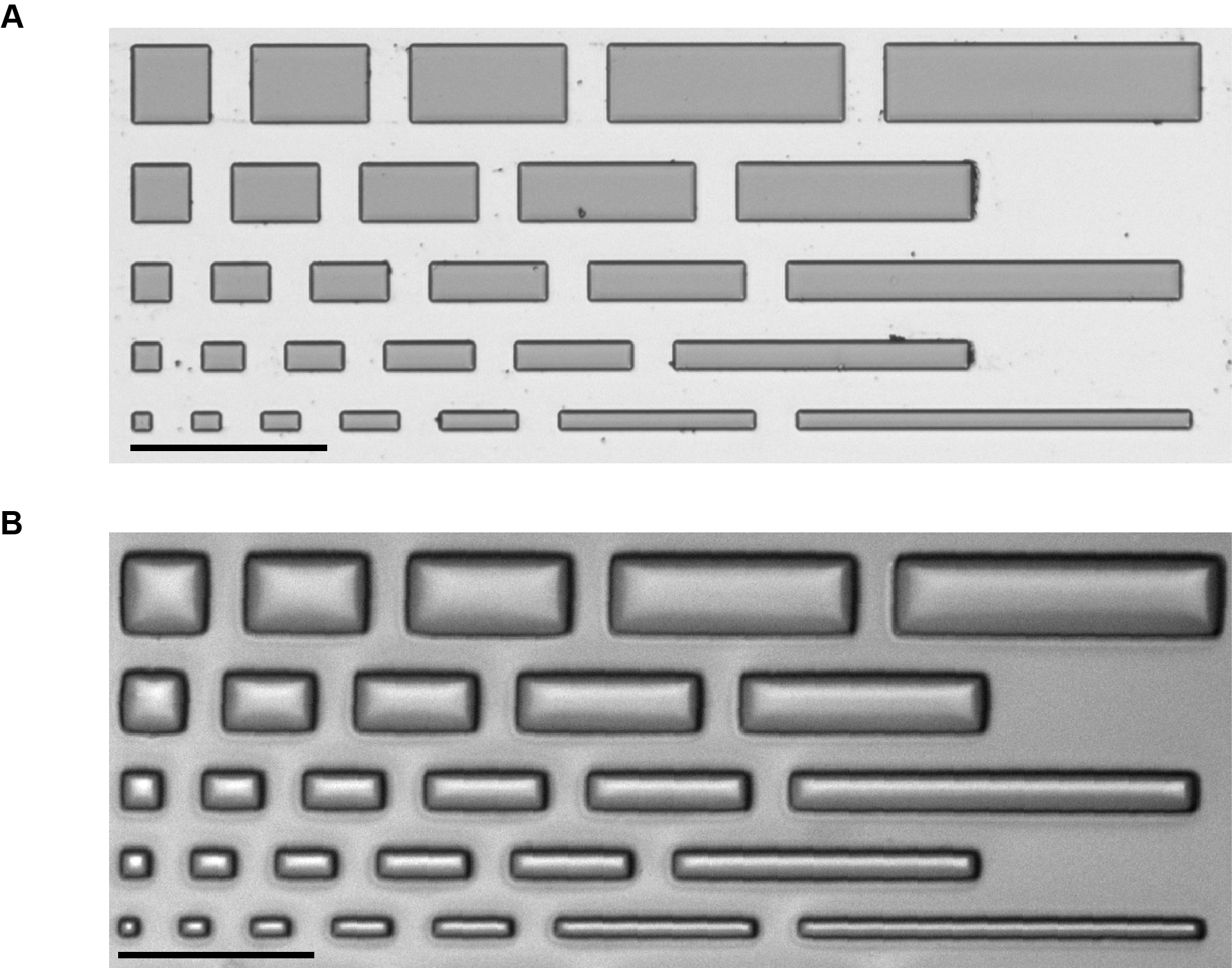

Supplement: Figure S2 — SU-8 pattern on a wafer and molded acrylamide gel. A) Image of a silicon wafer with 3 µm high patterns in SU-8 photoresist. B) Structures shown in A have been molded in a polyacrylamide gel. Scale bars 100 µm. The smallest features are 10 µm wide. (TIF) [file pone.0075537.s002.tif]

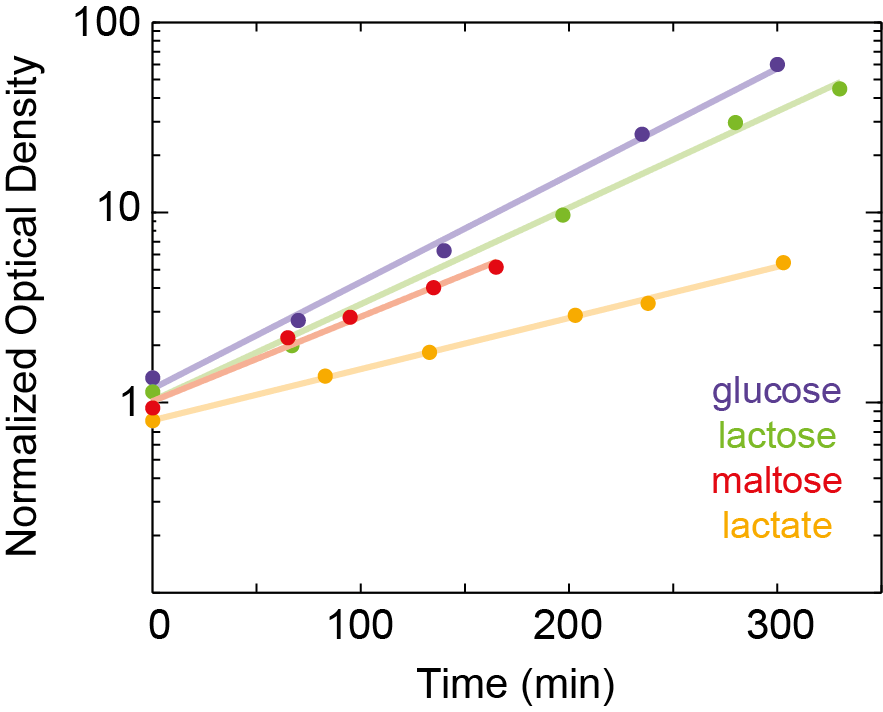

Supplement: Figure S3 — Growth rates in batch cultures. Optical density at 550 nm normalized by the OD at t=0 (and shifted for clarity) versus time for batch cultures of MG1655 cells growing in minimal medium with abundant (0.1%) glucose (dotted line), lactose (green), maltose (red) and lactate (yellow) as sole carbon source. Exponential fits to the experimental data points (lines) yielded growth rates of 1.12 h-1 on glucose, 1.01 h-1 on lactose, 0.88 h-1 on maltose and 0.54 h-1 on lactate, comparable to those obtained for cells growing in the microfluidic device (see main text and Figure 3B and 3D). (TIF) [file pone.0075537.s003.tif]
